# The Influence of Physical Exercise, Ketogenic Diet, and Time-Restricted Eating on De Novo Lipogenesis: A Narrative Review

**DOI:** 10.3390/nu17040663

**Published:** 2025-02-13

**Authors:** Antonio Paoli

**Affiliations:** 1 Department of Biomedical Sciences, University of Padua, 35100 Padua, Italy; antonio.paoli@unipd.it; 2 Research Center for High Performance Sport, UCAM Catholic University of Murcia, 30107 Murcia, Spain

**Keywords:** fat metabolism, ketone bodies, intermittent fasting, liposynthesis, MASLD, NALFD, liver, adipose tissue, endurance training

## Abstract

De novo lipogenesis (DNL) is a metabolic pathway that converts carbohydrates into fatty acids, primarily occurring in the liver and, to a lesser extent, in adipose tissue. While hepatic DNL is highly responsive to dietary carbohydrate intake and regulated by insulin via transcription factors like SREBP-1c, adipose DNL is more modest and less sensitive to dietary overfeeding. Dysregulated DNL contributes to metabolic disorders, including metabolic dysfunction-associated steatotic liver disease (MASLD). Lifestyle interventions, such as physical exercise, ketogenic diets, and time-restricted eating (TRE) offer promising strategies to regulate DNL and improve metabolic health. Physical exercise enhances glucose uptake in muscles, reduces insulin levels, and promotes lipid oxidation, thereby suppressing hepatic DNL. Endurance and resistance training also improve mitochondrial function, further mitigating hepatic triglyceride accumulation. Ketogenic diets shift energy metabolism toward fatty acid oxidation and ketogenesis, lower insulin, and directly downregulate lipogenic enzyme activity in the liver. TRE aligns feeding with circadian rhythms by optimizing AMP-activated protein kinase (AMPK) activation during fasting periods, which suppresses DNL and enhances lipid metabolism. The combined effects of these interventions demonstrate significant potential for improving lipid profiles, reducing hepatic triglycerides, and preventing lipotoxicity. By addressing the distinct roles of the liver and adipose DNL, these strategies target systemic and localized lipid metabolism dysregulation. Although further research is needed to fully understand their long-term impact, these findings highlight the transformative potential of integrating these approaches into clinical practice to manage metabolic disorders and their associated complications.

## 1. Introduction

De novo lipogenesis (DNL) is the metabolic pathway by which carbohydrates are converted into fatty acids and subsequently esterified to form triglycerides. This process primarily occurs in the liver but can also occur in adipose tissue and, to a lesser extent, in other organs. Although adipose DNL was historically considered of minimal importance [1,2], recent findings using deuterium-labeled water (D_2_O) have demonstrated that approximately 20% of palmitate in human triglycerides is derived from DNL, with significant interindividual variability (up to 50%) [3].

DNL plays a critical role in maintaining lipid homeostasis, energy storage, and overall metabolic balance and is strictly regulated by many factors. Under normal physiological conditions, DNL activity remains relatively low because dietary fat is the primary source of circulating lipids. However, excess carbohydrate intake, particularly when accompanied by elevated insulin levels, upregulates DNL, thereby increasing lipid synthesis and storage. While evolutionarily advantageous for storing surplus energy, chronic DNL activation can contribute to metabolic disorders. Indeed, a high-carbohydrate diet can, therefore, heavily supply the DNL pathway, driving an increase in DNL activity [4]. This upregulation leads to the accumulation of DNL products, specifically fatty acyl chains linked to coenzyme A, which integrate into various lipid species. These lipids can then serve additional metabolic roles, but also potentially result in adverse effects when DNL is elevated, contributing, for example, to the pathogenesis of metabolic dysfunction-associated steatotic liver disease (MASLD), previously known as non-alcoholic fatty liver disease (NAFLD) [5]. Moreover, DNL is significantly increased in patients with obesity, suggesting the reduction of DNL as one of the therapeutic approaches [6]. Considering the positive effects of physical exercise, fasting, and/or ketogenic diet on liver metabolism, it is possible to hypothesize that this effect may be related to the regulation of DNL; therefore, the aim of this review is to discuss the effects of physical exercise, fasting, and nutritional ketosis on DNL both in the liver and adipose tissue. A literature search was conducted using major biomedical databases, including PubMed (Medline) and Scopus, on December 31, 2024, without any restrictions on the year of publication. As this is a narrative review, we have included numerous studies that also discuss the physiological, molecular, and biochemical mechanisms underlying de novo lipogenesis.

## 2. Mechanisms of De Novo Lipogenesis

As mentioned previously, DNL is influenced by multiple factors, including hormonal regulation, nutrient availability, and the transcriptional control of key enzymes.

### 2.1. Hormonal and Transcriptional Regulation of DNL

Insulin is the primary hormone that drives DNL. In response to carbohydrate intake, insulin levels increase, stimulating glucose uptake and its conversion into acetyl-CoA in the liver. Insulin also upregulates key enzymes involved in lipogenesis, including acetyl-CoA carboxylase (ACC) and fatty acid synthase (FAS). ACC catalyzes the carboxylation of acetyl-CoA to form malonyl-CoA, which is the first committed step in fatty acid synthesis. FAS then uses malonyl-CoA as a substrate to elongate fatty acid chains, eventually producing palmitate, a 16-carbon saturated fatty acid [7]. Additionally, insulin suppresses lipolysis in adipose tissue, limiting the release of fatty acids and directing the body’s energy needs toward carbohydrate metabolism, further promoting DNL.

Conversely, glucagon, the counter-regulatory hormone of insulin, inhibits DNL. During fasting or carbohydrate restriction, glucagon levels increase, activating pathways that promote lipid oxidation over lipogenesis. Glucagon reduces the activity of ACC and FAS, and stimulates AMP-activated protein kinase (AMPK), an enzyme that inhibits ACC through phosphorylation, thereby suppressing the DNL pathway [8].

DNL is controlled at the transcriptional level by several key transcription factors including sterol regulatory element-binding protein-1c (SREBP-1c) and carbohydrate-responsive element-binding protein (ChREBP) [9]. Insulin promotes the activity of SREBP-1c, which increases the transcription of ACC and FAS, thereby enhancing DNL. SREBP-1c is an essential mediator of the lipogenic response to insulin and carbohydrate intake, and its upregulation is often observed in metabolic disorders characterized by hyperinsulinemia, such as obesity and type 2 diabetes [10,11,12].

ChREBP is activated in response to glucose and functions in concert with SREBP-1c to increase lipogenic gene expression. High levels of glucose metabolites, such as glucose-6-phosphate and xylulose-5-phosphate, activate ChREBP, which then translocates to the nucleus and binds to carbohydrate-responsive elements (ChREs) in the promoter regions of DNL genes, further enhancing the synthesis of fatty acids [13,14]. Together, SREBP-1c and ChREBP act as metabolic sensors that regulate the flux of carbohydrates into DNL, ensuring that excess carbohydrates are efficiently stored as fat.

Apart from insulin and glucagon, there are other hormones that influence DNL in both the liver and adipose tissue [15]. Growth hormone (GH) plays a key role in inhibiting DNL through mechanisms that are independent of classic insulin-mediated pathways. The loss of hepatic GH signaling in aLivGHRkd mice leads to increased DNL despite normal or low levels of mature SREBP-1c. Instead, GH appears to regulate DNL through its effects on glycolysis. First, GH suppresses glucokinase (GCK) expression, which otherwise promotes glycolysis by trapping glucose as glucose-6-phosphate. In GH-deficient mice, increased GCK activity facilitates glucose flux into glycolysis, enhancing DNL. Second, the loss of GH signaling leads to increased levels of fructose-2,6-bisphosphate (F2,6BP), an activator of phosphofructokinase-1, and an inhibitor of fructose-1,6-bisphosphatase, thereby promoting glycolysis and reducing gluconeogenesis. Third, GH upregulates ChREBP activity, as evidenced by the increased expression of its target genes Pklr and G6pc in GH-deficient mice. These findings suggest that GH inhibits hepatic DNL primarily by suppressing glycolysis at multiple control points, and that GH deficiency may contribute to NAFLD through increased glucose-driven lipid synthesis [16].

Thyroid hormones (TH) also regulate hepatic DNL through multiple pathways. Even though many effects of TH seem to stimulate DNL (direct stimulation of ACC and FAS expression through thyroid hormone receptors (THRs), upregulation of ChREBP, both up- and downregulation of SREBP-1c, increased glycolytic flux by upregulating GCK and other glycolytic enzymes), the net effect of TH is a reduction of TG in the liver and adipose tissue due to its effect on promoting TG oxidation [17]. New players have emerged in the regulation of DNL. Incretins are promising drugs for the control of DNL. A recent study demonstrated that Imeglimin (an oral antidiabetic agent) suppresses liver DNL by decreasing SREBP-1c and ChREBP while improving fatty acid oxidation in a high-fat, high-sucrose diet-fed mouse model [18].

### 2.2. Nutrient Availability and Substrate Provision

Carbohydrate availability is a key driver of DNL. High carbohydrate intake provides excess glucose, which is initially used for immediate energy production. Surplus glucose is directed into the pentose phosphate pathway, generating NADPH, a cofactor required for fatty acid synthesis. Concurrently, excess glucose is converted to acetyl-CoA via glycolysis, which is the primary building block for fatty acid synthesis. This acetyl-CoA enters the DNL pathway in the liver to synthesize fatty acids, particularly when the glycogen storage capacity is exceeded [7,19].

Fructose, a monosaccharide found in sucrose and high-fructose corn syrup, is also a potent stimulator of DNL. Unlike glucose, which is partially metabolized in extrahepatic tissues, fructose is almost exclusively metabolized in the liver, bypassing certain regulatory steps. As a result, fructose promotes rapid accumulation of acetyl-CoA and an increase in hepatic DNL activity, as demonstrated by an increase in palmitate studied with tracers [20].

This scenario may be worsened by excessive energy intake. During the postprandial period, there is reduced suppression of adipose tissue lipolysis, with subsequently elevated levels of plasma non-esterified fatty acids (NEFAs). These NEFAs are then absorbed by the liver. When dietary fat intake is excessive, fatty acids released from chylomicron triglycerides (TAGs) contribute to the plasma NEFA pool through a process known as chylomicron-TAG “spillover”, which can also result in increased hepatic NEFA uptake.

Hyperinsulinemia further enhances hepatic glucose uptake and activates DNL through mechanisms involving sterol regulatory element-binding protein-1 (SREBP-1), As, and ACC. However, DNL is not limited to the liver; indeed, it has been demonstrated in humans that it also takes place in adipose tissue after meals [21]. Furthermore, it has been demonstrated very recently, although in a mouse model, that excess protein could increase the risk of MASLD via the metabolism of amino acids. Indeed, in vivo, dietary amino acids are twice as efficient as glucose in fueling hepatic fatty acid synthesis [22].

### 2.3. DNL in Liver and Adipose Tissue

As stated before, the main target of DNL is the liver; adipose tissue, although involved, is less influenced, for instance, by nutritional aspects. Two decades ago, Diraison and colleagues [23] examined the differences in the regulation of DNL by carbohydrates in human adipose tissue and the liver in vivo, comparing the responses of hepatic and adipose tissue lipogenesis to acute oral glucose ingestion and a two-week high-carbohydrate (HC), high-energy diet in healthy subjects.

Using deuterated water and [U-13C] acetate as tracers, along with measurements of mRNA levels of lipogenic enzymes and SREBP-1c in adipose tissue, the study revealed clear differences in the regulation of lipogenesis between the liver and adipose tissue in response to carbohydrates. Quantitative estimates have shown that hepatic lipogenesis contributes significantly to TAG synthesis. In adipose tissue, quantitative estimates suggested that “adipose DNL was minimal (<1 g over 12 h) and poorly stimulated by oral glucose or the HC diet”. These results, along with those from other authors [3], confirmed that DNL in human subjects is active, although highly variable and present in low amounts (from 20 to 50%) [24], and that DNL in adipose tissue is less responsive than hepatic lipogenesis to acute and/or prolonged carbohydrate overfeeding.

It is noteworthy that the regulation of lipogenesis by carbohydrates differs significantly between humans and other species. In humans, adipose tissue exhibits relatively low lipogenic activity and a lower responsiveness to carbohydrates and insulin compared to the liver. In contrast, in species such as rats, adipose tissue lipogenesis is more robust and highly responsive to these stimuli. This reduced responsiveness in human adipose tissue may be partly attributed to the limited stimulation of SREBP-1c expression, a key regulator of lipogenic pathways. The low expression of SREBP-1c in human adipose tissue may contribute to this poor responsiveness.

### 2.4. The Specific Role of High-Carbohydrate Diet and Fructose: Not All Carbohydrates Are the Same

A deeper exploration of the role of high-carbohydrate diets and fructose consumption and their distinct effects on DNL in the liver and adipose tissue is necessary. The liver, the primary site of fructose metabolism, plays a pivotal role in its conversion to lipogenic substrates. Fructose bypasses the tightly regulated glycolytic pathway by entering metabolism through fructokinase and aldolase B, leading to the accumulation of trioses and subsequent stimulation of DNL. As previously stated, this pathway enhances the synthesis of fatty acids by upregulating the activity of key enzymes like FAS and ACC. Notably, the consumption of fructose-sweetened beverages has been shown to double the hepatic fractional secretion rate of newly synthesized palmitate, a primary lipogenic fatty acid, in comparison with glucose or control groups, even without excess caloric intake [20].

In the context of adipose tissue, fructose does not directly stimulate DNL to the same extent as it does in the liver. Instead, it may indirectly contribute to adipose fat deposition through the increased delivery of very-low-density lipoprotein (VLDL)-triglycerides synthesized in the liver. Adipose tissue lipolysis and FFA flux remain largely unchanged under moderate fructose intake, as observed by Geidl-Flueck and colleagues [20]. However, chronic high-carbohydrate diets can elevate circulating insulin levels, suppressing lipolysis and promoting fat storage in adipose tissue. This differential impact underscores the liver’s dominant role in fructose-induced lipogenesis compared to adipose tissue.

High-carbohydrate diets, including those rich in fructose, also influence hepatic substrate flux and lipogenic gene expression. Persistent carbohydrate intake enhances hepatic glucose uptake and its conversion into lipogenic precursors via glycolysis and pentose phosphate pathway. This process is amplified in the presence of fructose due to its ability to activate glucokinase and increase glycolytic intermediate availability, creating a synergistic effect when consumed with glucose as sucrose [25]. The result is a metabolic adaptation characterized by elevated basal hepatic DNL capacity. This adaptation is hypothesized to prepare the liver for recurrent high-carbohydrate loads, but also poses risks of developing MASLD and systemic insulin resistance over time.

Moreover, the metabolic repercussions of fructose extend beyond its direct lipogenic effects. The production of short-chain fatty acids like acetate from microbiota fermentation of dietary fructose further fuels hepatic DNL [26]. This interaction between fructose metabolism and gut-derived metabolites highlights a complex network contributing to hepatic lipid overload [27]. While adipose tissue primarily serves as a storage depot, the liver’s imbalance between lipogenesis and oxidation under high-fructose and high-carbohydrate conditions underscores its central role in the pathogenesis of metabolic diseases.

## 3. Role of DNL in Energy Storage and Metabolic Health

DNL plays an essential role in energy storage by converting surplus dietary carbohydrates into long-term fat storage. In ancestral human populations, this adaptation may have provided an evolutionary advantage during times of food scarcity [28,29]. However, in modern societies, where food is abundant, chronic upregulation of DNL due to excessive carbohydrate consumption can lead to excessive fat storage, particularly in the liver and adipose tissue. Moreover, some researchers have suggested a parallel between the consumption of fructose (raised in the last decades [30,31]) and its fermentation byproduct, ethanol; indeed, both serve as substrates for DNL, and their consumption is related to hedonic factors [32].

The role of DNL in MASLD has been extensively studied, with research indicating that approximately 26% of hepatic triglycerides in individuals with MASLD are derived from DNL [6]. Elevated DNL contributes to an increase in hepatic triglyceride content, promoting liver steatosis, inflammation, and ultimately fibrosis [33]. Additionally, increased lipogenesis has been linked to higher production of very-low-density lipoprotein (VLDL), which can contribute to dyslipidemia and increase the risk of cardiovascular disease [34,35].

## 4. Regulation of DNL by Exercise, Ketogenic Diet, and Time Restricted Eating

Given the link between DNL and metabolic diseases, lifestyle interventions, such as physical activity, carbohydrate restriction (as in ketogenic diets), and intermittent fasting, have gained attention for their potential to downregulate DNL and improve metabolic health. Physical activity reduces substrate availability for DNL by increasing glucose uptake in muscle tissue, thereby lowering blood glucose and insulin levels [36]. Similarly, ketogenic diets limit carbohydrate intake and stimulate lipolysis, which shifts the body toward fat as the primary energy source and suppresses DNL [37,38,39]. Intermittent fasting cycles modulate insulin and glucagon levels, favoring lipid oxidation over lipogenesis during fasting periods, which can reduce hepatic lipid accumulation and improve metabolic flexibility [40].

### 4.1. Physical Exercise and De Novo Lipogenesis

#### 4.1.1. Physical Exercise: Define the Context

First, it is essential to define physical activity and its subcategories.

(a) Physical activity: This refers to any effort exerted by the musculoskeletal system that results in higher energy expenditure than at rest (e.g., walking, climbing stairs, and housework).
(b) Physical exercise: A subcategory of physical activity characterized by being planned, structured, repetitive, and purposeful, aiming to improve or maintain one or more components of physical fitness (e.g., running, gym workouts, and swimming).
(c) Sports activity: This encompasses the first two categories but occurs within competitive or non-competitive contexts governed by structured rules. It is codified in a manner that ensures recognition through standardized rules and mechanisms applicable in both official and unofficial settings [41,42].

This paper will explore the effects of physical exercise (PE)—the planned, structured, and purposeful form of physical activity—on DNL. However, before delving into these effects, it is crucial to outline the primary types of PE, namely endurance training (ET) and resistance training (RT).

Endurance training (ET) typically involves large muscle groups performing cyclic repetitive movements over an extended period at moderate intensities. This type of exercise emphasizes aerobic energy production and fosters cardiovascular and muscular endurance. ET can vary based on factors such as intensity (e.g., speed), volume (e.g., distance covered), and type of movement (e.g., running, rowing, cycling, and swimming).

Resistance training (RT), on the other hand, encompasses a variety of exercises aimed at inducing muscular adaptations and enhancing strength by working against resistance or load. RT includes traditional weightlifting with barbells or dumbbells, bodyweight exercises such as push-ups, and other forms. The common aim is to cause muscular adaptations and increase strength by working against resistance or loads [43]. RT exercises are defined by several important variables, including muscle action used, type of resistance applied, volume (total number of sets and repetitions), exercise selection and workout structure (e.g., the number of muscle groups trained), sequence of exercises performed, rest intervals between sets, repetition speed, and training frequency [44,45,46].

#### 4.1.2. Effects of Physical Exercise on DNL

Physical exercise has a significant impact on lipid metabolism. Most notably, ET promotes the oxidation of circulating lipids and utilization of fat stores both during and after exercise in a chronic manner [47]. Furthermore, ET improves insulin sensitivity and glucose utilization, thereby reducing lipogenesis. RT also influences lipid metabolism, albeit with a delayed effect, by increasing energy expenditure after exercise.

During exercise, particularly ET, adipose tissue releases NEFA into the bloodstream via lipolysis. Concurrently, exercise training promotes NEFA uptake by skeletal muscles, reducing their availability to the liver. Increased lipoprotein lipase (LPL) activity in skeletal muscle further enhances the uptake of chylomicron-TAGs, limiting hepatic NEFA flux. Moreover, exercise increases fat oxidation during the post-exercise period. Indeed, given that energy provision at rest relies predominantly on fat oxidation, the increase in energy expenditure for a few hours after exercise, known as Excess Post-Exercise Oxygen Consumption (EPOC), enhances the clearance of fatty acids from the bloodstream [48,49].

While the specific impact of exercise training on DNL in humans has not been directly studied, evidence from animal models offers a detailed understanding. Studies in rats genetically predisposed to develop type 2 diabetes and obesity (OLETF) show that exercise reduces hepatic fat storage by suppressing the activity of key enzymes in the DNL pathway, including acetyl-CoA carboxylase (ACC) and fatty acid synthase (FAS) [50,51]. This suppression correlates with reductions in fasting glucose and insulin levels, both of which are central to regulating DNL. Elevated insulin and glucose levels stimulate DNL by driving glucose flux through glycolysis and acetyl-CoA production, which is the precursor for fatty acid synthesis. Indeed, exercise training lowers plasma insulin, which in turn reduces DNL activity. This effect is supported by animal studies that show a decline in ACC and FAS activity [52,53].

Moreover, exercise in animal models has been linked to lower intrahepatic triglyceride content, increased PGC-1α expression, and higher mitochondrial protein levels associated with mitochondrial function, such as cytochrome c (Cyt c) [54], β-hydroxyacyl-CoA dehydrogenase (β-HAD) [55], and citrate synthase (CS) [56]. These effects seem to be mediated by different pathways and mediators, such as Irisin [57] and Fibroblast Growth Factor 21 [55], among others.

Exercise also influences the previously mentioned hepatic lipogenic transcription factor sterol regulatory element-binding protein 1c (SREBP-1c). Notably, in strength-trained mice, the expression of SREBP-1c and its downstream targets—such as ACC, stearoyl-CoA desaturase 1 (SCD1), FAS, and glycerol-3-phosphate acyltransferase (GPAT1)—was significantly reduced compared to their sedentary counterparts [58]. Moreover, elevated phosphorylation of AMP-activated protein kinase (AMPK) and SIRT1 in trained groups enhances hepatic lipid oxidation and lipophagy, fostering a balanced lipid metabolism [59]. Intense exercise also plays a crucial role in blunting the DNL during overfeeding. In one study [60], regular vigorous exercise was shown to reduce markers related to DNL in human adipose tissue, even in the presence of an energy surplus. Indeed, vigorous exercise prevents the reduction of insulin sensitivity, development of hyperinsulinemia, and altered expression of numerous genes caused by reduced physical activity and short-term overfeeding, which ultimately regulate DNL.

In humans, indirect evidence supports these findings. Both endurance and resistance training positively affect glucose metabolism, lower fasting glucose and insulin concentrations, enhance whole-body insulin sensitivity [61], and reduce intrahepatic triglyceride (IHTG) content [62]. These effects are more pronounced in response to meals, as training improves postprandial insulin dynamics, further limiting the substrates required for DNL.

Despite this strong correlation, direct studies measuring DNL activity in humans before and after exercise training are needed to confirm its role in reducing intrahepatic triglyceride content. Such studies could deepen our mechanistic understanding of how exercise improves hepatic and systemic metabolic health, bridging the gap between animal and human data.

### 4.2. Ketogenic Diet and De Novo Lipogenesis

#### 4.2.1. Physiological Bases of a Ketogenic Diet

The ketogenic diet is a high-fat, low-carbohydrate, and moderate-protein dietary strategy that induces a metabolic state known as ketosis. In this state, the body shifts to primarily using fat as its energy source [63]. Originally referred to as a “protein-sparing modified fast” [64], the ketogenic diet was first employed as a medical treatment by Dr. Wilder in 1921 as an alternative to complete fasting for managing epilepsy [65]. The term “protein-sparing” highlights the goal of achieving ketosis—a state usually triggered by fasting—without excessive protein breakdown. In 1926, the first structured 4:1 ketogenic diet protocol was introduced, prescribing 1 g of protein per kg of body weight daily, 10–15 g of carbohydrates per day, and the remainder of calories from fats, maintaining a 4:1 ratio of fat to combined carbohydrates and protein [66]. This framework has remained consistent over time, with minor modifications, generally limiting carbohydrate intake to less than 30–50 g or about 5% of the total daily energy consumption [38,39,67]. Recently, a clear classification of different types of ketogenic diets has been published [68]. Over the years, interest in the ketogenic diet has expanded beyond its original use in epilepsy to address a range of other conditions, including inflammation [69], metabolic syndrome, type 2 diabetes [70], polycystic ovary syndrome [71], cancer [72,73], and other non-communicable diseases [39]. This dietary approach is gaining prominence as a public health intervention due to its ability to rapidly address modifiable risk factors, particularly those linked to obesity and its associated metabolic complications [74].

The proposed health benefits of a ketogenic diet are rooted in the concept of “physiological ketosis”, first described by Hans Krebs in 1966 [75]. Unlike “pathological ketosis”, which occurs in conditions such as uncontrolled diabetes and is characterized by low systemic pH, extremely low insulin levels, hyperglycemia, and ketone body levels exceeding 7–8 mmol/L, physiological ketosis maintains normal pH with ketone body levels typically ranging between 0.3 and 4 mmol/L [76]. During carbohydrate restriction, ketone bodies are synthesized in the liver through ketogenesis. This process involves the conversion of acetyl-CoA into acetoacetyl-CoA via acetoacetyl-CoA thiolase, followed by the formation of 3-hydroxy-3-methylglutaryl-CoA (HMG-CoA) catalyzed by HMG-CoA synthetase. HMG-CoA lyase then converts HMG-CoA to acetoacetate, which can be further reduced to β-hydroxybutyrate or spontaneously decarboxylated to acetone. These ketone bodies are more than mere byproducts of carbohydrate restriction; they act as active metabolic agents influencing various pathways [77]. Notably, the reduction in circulating insulin and the activation of FFA oxidation reduce the metabolic windmill of DNL and the activity of DNL-related enzymes.

#### 4.2.2. Ketogenic Diet and DNL

Most studies on KD and DNL have investigated the effects of a very low carbohydrate diet on hepatic DNL. Indeed, in recent times, KD has emerged as an effective nutritional strategy for the management of MASLD, and many studies have shown a rapid and marked reduction in liver fat accompanied by significant decreases in body weight in patients with MASLD treated with KD [78,79,80,81,82]. One study reported a greater reduction in liver TG (by ~55%) (with a concurrent reduction in body weight (−4.6 kg)) in obese subjects with NAFLD after two weeks of KD compared to caloric restriction alone. While the weight reduction was similar in the two groups (−4.6 ± 1.5 kg in the KD group vs. −4.0 ± 1.5 kg in the calorie-restricted group) [83], the liver TG reduction was more pronounced in the KD group. Perhaps the most outstanding results are those reported by Lukkonen et al., who observed a 31% decrease in IHTG content in just six days of KD, despite only a 3% reduction in body weight. in 10 overweight individuals with obesity [80].

The beneficial effects of KD on hepatic DNL can be attributed to several mechanisms.

1. KD decreases insulin levels and increases the FA oxidation rate, leading to a subsequent reduction in lipogenesis [84,85] in all tissues, including the liver.
2. Extreme carbohydrate reduction (below 30/50 g CHO per day) stimulates AMPK and SIRT1, even in the absence of caloric restriction. The activation of both SIRT1 and AMPK impacts glucose homeostasis mainly by improving insulin sensitivity [86]. It should be noted that, in mainstream media and on the Internet, low-carb diets are often confused with ketogenic diets. While the former primarily operates by reducing caloric intake through carbohydrate restriction and inducing a slight decrease in insulin production, the latter induces a specific metabolic state, ketosis, which exerts its effects through various complex metabolic pathways [63], not merely those associated with mere caloric restriction [38].
3. As shown by Luukkonen et al. [80], the reduction in IHTG can be attributed to increased net TG hydrolysis and partitioning of the resulting fatty acids toward ketogenesis (+232%), driven by reductions in serum insulin concentrations (−53%) and hepatic citrate synthase flux (−38%), ultimately suppressing DNL.
4. Additionally, an increased hepatic mitochondrial redox state (+167%) was observed, suggesting that hepatic mitochondrial activity may represent a potential treatment target in NAFLD [80].
5. As observed in animal models, KD exerts anti-steatogenic effects by enhancing the hepatic expression of key genes involved in mitochondrial biogenesis and fatty acid oxidation (PGC-1α and FGF21), while suppressing inflammatory genes (TNF-α, Nf-kb, and Il-6) [87]. Indeed, using a multi-omics approach, Mardinoglu and colleagues demonstrated that improvements in liver fat metabolism in individuals with obesity and NAFLD following a short-term intervention with an isocaloric KD (averaging 3115 kcal/day) were characterized by a rapid decline in many inflammatory markers (e.g., IL-6, TNF-α).
6. The stimulation of DNL by inflammation, particularly in the liver, has been well documented since the 1990s [88], as well as in adipose tissue [89]. A recent paper by Guo et al. [90] using transcriptional analysis demonstrated that KD improves hepatic steatosis via FGF21 and its receptor klotho (KLB) pathway activation in a murine model, confirming the crucial role of the FGF21 pathway in hepatic fat reduction during KD [91]. However, in humans, short-term fasting and ketogenic diet consumption do not significantly alter circulating FGF21 levels. Instead, fructose intake induces a rapid and substantial increase in FGF21 levels, which gradually return to baseline within a few hours [13].

It is crucial to note that in most studies, there was no control group, and KD was combined with caloric restriction. Since KD, caloric restriction, and fasting share several metabolic pathways and targets, a potential synergistic effect cannot be excluded [92].

Regarding the relationship between the ketogenic diet (KD) and DNL in adipose tissue, limited studies are available, primarily based on animal models. One study demonstrated that KD limits glycolytic substrate availability, thereby inhibiting mTORC1 activation and maintaining DNL suppression, even in the presence of Insig1 overexpression (a negative regulator of SREBP transcriptional activity). These findings suggest that KD’s effects on adipose tissue DNL are primarily mediated by substrate limitation. [93].

The direct role of BHB on DNL is less clear. Nishitani and colleagues reported that BHB enhances DNL in adipocytes by upregulating the expression of PPARγ and lipogenic genes, but this effect is dependent on the presence of insulin. In the absence of insulin, BHB did not activate these pathways, indicating that its role in DNL is closely linked to insulin signaling. Thus, BHB, in conjunction with insulin, may promote lipid accumulation and support adipose tissue function in the post-fasting state [94].

In male Wistar rats, KD was found to elevate glycerol kinase activity, favoring TAG recycling in white adipose tissue WAT), which increased uncoupling protein-1 levels in brown adipose tissue (BAT). This suggests that KD preserves insulin sensitivity, maintains lipolytic capacity in WAT, and enhances thermogenesis [95]

In humans, indirect evidence suggests that KD reduces lipogenic pathways, as demonstrated by a decrease in circulating palmitoleic acid (16:1n-7), a product of Δ9 desaturase. Palmitoleic acid is a minor constituent of dietary fat and is associated with higher adiposity. Notably, researchers reported that despite a threefold increase in dietary saturated fat intake during KD, circulating palmitoleic acid levels significantly declined, highlighting the diet’s capacity to suppress lipogenesis under these conditions. [96].

Two important aspects deserve attention: the effect of a ketogenic diet (KD) on blood lipids (given its high fat intake) and the metabolic changes that occur upon discontinuing KD. Regarding lipid profiles, cholesterol homeostasis is tightly regulated by the balance between synthesis, uptake, and excretion to fulfill cellular demands while preventing excessive accumulation. Insulin plays a pivotal role in this regulation by influencing key enzymes and transport mechanisms. For instance, in the liver, insulin promotes the expression of sterol regulatory element-binding protein-2 (SREBP-2), a transcription factor that upregulates 3-hydroxy-3-methylglutaryl-CoA reductase (HMG-CoA reductase), the rate-limiting enzyme in cholesterol biosynthesis. [97]. The impact of KD on lipid parameters remains debated, with meta-analyses yielding mixed findings. In individuals with overweight and obesity, KD showed no significant differences in lipid profiles compared to low-calorie diets [98]. Conversely, in normal-weight individuals, a meta-analysis reported increased total and LDL cholesterol levels offset by an increase in HDL cholesterol [99]. When compared to low-fat diets, KD resulted in a reduction in triglycerides (TAG) and an increase in both LDL cholesterol and HDL cholesterol [100]. These findings highlight the nuanced effects of KD on lipid metabolism, which may vary depending on baseline body weight and dietary comparisons.

The transition from a ketogenic regimen to a normal diet has not been extensively studied. However, research has demonstrated that two 20-day periods of a ketogenic Mediterranean diet with phytoextracts (KEMEPHY), interspersed with six months of a balanced Mediterranean diet, resulted in significant reductions in body weight and fat mass. This suggests that a carefully managed transition from a ketotic state to a carbohydrate-rich diet can support weight loss [101]. Regarding appetite regulation after discontinuing a ketogenic diet, Sumithran and colleagues [102] observed that changes in peripheral hormones involved in appetite control persisted for up to one year following a very low-calorie diet. Their study reported reductions in anorexigenic hormones, including leptin, peptide YY, cholecystokinin, insulin, and pancreatic peptide, along with an increase in orexigenic hormone ghrelin. Notably, hunger levels remained elevated one year after ceasing the diet. In a similar experimental setting, the same group demonstrated that a ketogenic diet could suppress the ghrelin increase typically triggered by low energy intake [103].

### 4.3. Fasting and De Novo Lipogenesis

#### 4.3.1. Cultural and Historical Foundations of Fasting

Anthropologists, ethnologists, and historians of religion agree that fasting is a transversal phenomenon in all civilizations and human cultures, from East to West and North to South [104]. Even though the practice of fasting has endured throughout the history of mankind, its meaning has changed over the centuries. In a more ancient context, fasting was used as a ritual of symbolic death and rebirth before important “rites of passage” such as for kings, medicine men, or even the transition from childhood to adult life [104]. On these occasions, fasting was perceived as a cleansing procedure to prepare the individual to receive sacred forces or to reinforce themselves with sacred power [105]. More recently, in the universal religions framework, fasting became an expression of humility and/or penitence toward the deity. With the widespread affirmation of Christianity and Islam (with Ramadan), this meaning of fasting has become more common, related to the dichotomy of body and soul; thus, fasting was one of the ascetic practices that allowed the believer to “overcome” the body. Fasting is also related to the soul’s discipline, which helps the believer remain focused on prayer and religious quests without being distracted by food’s attractiveness [106]. The importance given to fasting in almost all religious traditions may probably be traced back to fasting/starvation periods embedded in pre-agrarian cultures during food shortages or famine periods [107]. The subjective experience of an expanded, lighter body and ecstasy (a Greek word meaning “to stand outside oneself”) may have led to the idea of transposing this experience of “self-transcendence” involving the dissolution of boundaries between the inner and outer body and mind into the body of religious practices [108]. Apart from religious and spiritual reasons, fasting has become a health practice (although even in sacred scriptures, there were clues about the positive health effects of religious fasting) in many cultures, as demonstrated by the works of Abū ʿImran Mūsā ibn ʿUbayd Allāh ibn Maymūn (aka Moses Maimonides 1135–1204) [109] and Luigi Cornaro (1484–1566) [110], where the practice of periodic fasting was described as one of the pillars of good health. At the end of the nineteenth century, physicians believed that fasting might favor infections; however, in 1899, Meltzer and Norris disproved this theory through an experiment using a dog model of infection [111]. A few years later (1904), the first scientific manuscript on the therapeutic use of fasting was published [112]. Since then, fasting as a form of therapy has started to attract growing interest [113]. More recently, fasting and periodic fasting have experienced a “renaissance” outside of the religious boundaries in a so-called “*secularisation of spiritual practices*” [108]. An often-cited example of fasting’s positive health effects may be found in the Gospel of Matthew, in the episode of the epileptic (demoniac) boy: “*… Howbeit this kind goeth not out but by prayer and fasting*” (*Matthew 17:14–21*). In this particular case, the therapeutic effect of fasting relies on ketosis (increase in ketone bodies in the bloodstream) induced by abstinence from food; indeed, in the 1920s, it was discovered that ketosis has positive effects on epileptic seizures [65]. In recent years, fasting has been In recent years, fasting has been “rediscovered” beyond traditional religions and employed in broader spiritual contexts as well as in a health-oriented approach.

#### 4.3.2. Time-Restricted Eating: An Age-Old Practice with a Modern Perspective

Time-Restricted Eating is a type of short-term fasting that can be included under the umbrella term of intermittent fasting (IF); it describes a nutritional approach that allows participants to consume food (with no specific indications about quantity) only within a defined window each day. TRE should ideally include a fasting period of at least 12 h; however, more options are available [63,92,114]. Considering the length of the eating and fasting windows, TRE protocols can be categorized as 16:8, 14:10, and 18:6 (fasting: eating ratio) [115]. Although the attention given to TRE has been relatively recent, a similar protocol has been practiced for centuries. Indeed, Ramadan fasting can be considered a form of time-restricted eating (TRE) as it prohibits food consumption during daylight hours.

However, several unique factors distinguish Ramadan fasting from other TRE practices. First, fasting and eating periods are inverted relative to the body’s natural circadian rhythm, as food is consumed after sunset and before sunrise. Second, the fasting window’s duration varies depending on the geography and season, as Ramadan follows the lunar calendar. Additionally, practices during Ramadan may differ; while some individuals eat both before sunrise and after sunset, others limit their intake to post-sunset meals only.

Apart from Ramadan, particular attention has been drawn lately in the scientific literature to the study of intermittent fasting, especially time-restricted feeding (TRF in animal models) [116,117,118], more accurately referred to as time-restricted eating (TRE) in humans [119,120,121,122].

Time-restricted eating (TRE) protocols induce a series of key metabolic adaptations that characterize fasting periods. These include maintaining blood glucose levels within the low-normal range, depleting or reducing glycogen stores, mobilizing fatty acids, and producing ketones. Additionally, TRE often leads to a decrease in circulating leptin levels and an increase in adiponectin levels, hormones that play critical roles in energy homeostasis and metabolic health [123,124]. These physiological changes are accompanied by behavioral shifts, such as heightened alertness and improved mental acuity during fasting periods [125].

#### 4.3.3. Time-Restricted Eating and DNL

It has been shown that alternate-day fasting alleviates high-fat diet-induced NAFLD in a mouse model through the activation of peroxisome proliferator-activated receptor alpha (PPARα), which, in turn, regulates fibroblast growth factor 21 (FGF21) expression [126]. Fibroblast growth factor 21 (FGF21) has diverse metabolic effects. Initially identified as a modulator of glucose uptake in adipocytes, its role has expanded to include the regulation of various metabolic pathways and a wide range of pharmacological benefits in animals and humans with metabolic dysfunction. Rather than directly influencing insulin secretion or appetite, FGF21 primarily facilitates lipid metabolism by promoting lipolysis, fatty acid oxidation, mitochondrial oxidative processes, and thermogenic energy expenditure. It is also essential for initiating and coordinating adaptive responses to starvation. Clinical studies have demonstrated that FGF21 improves dyslipidemia, reduces body mass, and enhances fasting insulin and adiponectin levels, indicating its potential as a therapeutic agent for metabolic disorders. FGF21 enhances hepatic insulin sensitivity, suppresses lipogenesis, DNL stimulates fatty acid β-oxidation, alleviates endoplasmic reticulum (ER) stress in the liver, and reduces very-low-density lipoprotein (VLDL) delivery to hepatocytes by downregulating VLDL receptor expression [127]. The effects of TRE on PPARa and FGF21 are similar to those of alternate-day fasting [33,128], and this effect is also mediated by the slight increase of BHB during the fasting period in TRE [129].

TRE activates AMPK, which, in turn, as discussed before, regulates lipid metabolism, including DNL. Indeed, AMPK activation occurs under conditions of energy deficit, such as fasting, where it promotes catabolic processes to restore energy homeostasis and simultaneously suppresses anabolic pathways like DNL in hepatocytes; AMPK downregulates SREBP-1c, thereby reducing the expression of enzymes such as acetyl-CoA, carboxylase (ACC), and fatty acid synthase (FASN) [130]. This suppression curtails the synthesis of fatty acids and triglycerides, which are key components of DNL.

TRE has demonstrated the potential to synchronize metabolic processes with circadian rhythms, amplifying its effects on lipid metabolism. Studies have suggested that TRE optimizes the diurnal activity of AMPK, which peaks during fasting periods, thereby enhancing lipid oxidation and suppressing lipogenesis during the fasting window, at least in mouse adipocytes [131]. Indeed, in animal models, TRE has been shown to significantly reduce hepatic triglyceride content and improve lipid profiles by modulating AMPK activity, even in the context of high-fat diets [59,132]. Furthermore, TRE-mediated AMPK activation has been associated with reduced endoplasmic reticulum stress and inflammation, which are often linked to dysregulated lipogenesis and the progression of metabolic diseases like non-alcoholic fatty liver disease (NAFLD) [133].

Although there is a lack of human studies directly investigating the effect of TRE on AMPK, clinical data have confirmed the positive effects of TRE on steatosis in mitigating excess DNL [134,135], presumably through AMPK-related pathways. Moreover, the alignment of TRE with natural circadian rhythms enhances metabolic efficiency, as key enzymes involved in lipid metabolism exhibit time-dependent activity, improve fat oxidation, and reduce lipogenesis not only in animal models but also in humans [136]. Moreover, it has been demonstrated that TRE increases BDNF, which is not only beneficial for hippocampal function but also closely linked to AMP-activated protein kinase (AMPK) activity. Increased expression of BDNF mRNA has been associated with enhanced fat oxidation through an AMPK-dependent mechanism, both in vivo and in vitro. This effect appears to be mediated primarily within skeletal muscle, potentially through autocrine and/or paracrine signaling pathways [137].

## 5. Conclusions

**Figure 1:**
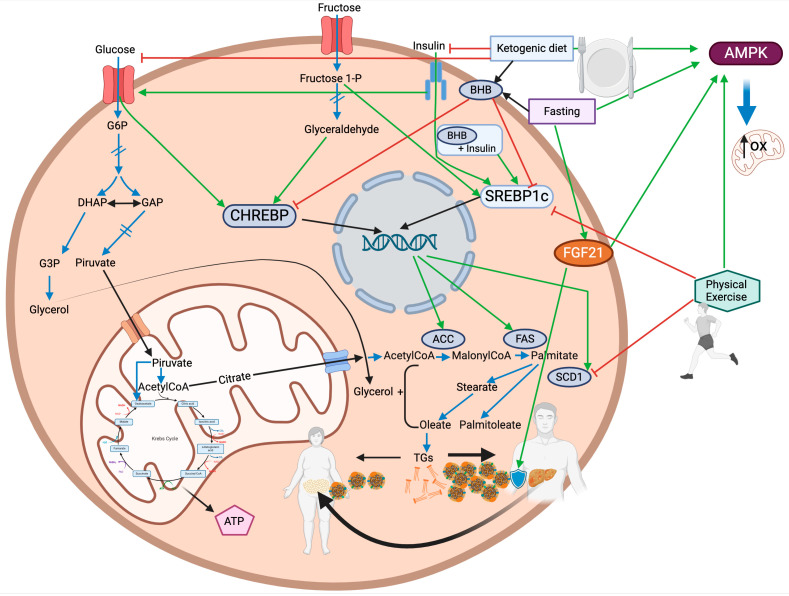
Effects of a ketogenic diet, fasting, and physical Exercise on DNL in both the liver and adipose tissue. The blue arrow indicates a single metabolic step. The blue double-bar arrow indicates multiple metabolic steps. The black arrow indicates the movement of the molecule throughout the cell/organelles. The green arrow indicates a positive stimulating effect. The red lines ending with perpendicular bars indicate inhibition. See the text for further details. Created using BioRender.com (accessed on 4 February 2025).

The interplay between lifestyle interventions, such as physical exercise, ketogenic diets, and time-restricted eating, demonstrates a profound influence on the regulation of DNL (Figure 1). These strategies collectively modulate key metabolic pathways, including the suppression of lipogenic enzymes, activation of lipid oxidation mechanisms, and enhancement of mitochondrial function. DNL differs significantly in its activity and regulation between the liver and adipose tissue.

In the liver, DNL plays a central role in converting excess carbohydrates into fatty acids, contributing to triglyceride synthesis and storage. This process is highly responsive to dietary intake, particularly carbohydrates, and is regulated by insulin through the activation of key transcription factors such as SREBP-1c.

DNL in adipose tissue is relatively limited and less responsive to dietary carbohydrate overfeeding. Adipose DNL primarily occurs under conditions of energy surplus and is driven by local substrate availability and lipogenic enzyme activity, which are less pronounced than those in the liver. These differences highlight the liver’s primary role in systemic lipid regulation and the more localized, modest contribution of adipose tissue to lipid synthesis. Moreover, the differential regulation of DNL between the liver and adipose tissue is significant in the context of metabolic health. Elevated hepatic DNL contributes to triglyceride accumulation within hepatocytes, a hallmark of MASLD. Conversely, the limited capacity of DNL in adipose tissue suggests that other pathways, such as dietary fat uptake and re-esterification of circulating fatty acids, play more substantial roles in adipose fat storage. Physical exercise, both endurance and resistance training, effectively reduces substrate availability for DNL by enhancing glucose uptake in the muscle tissue and lowering circulating insulin levels. This suppresses hepatic DNL while promoting lipid oxidation and mitochondrial biogenesis, which collectively reduces hepatic triglyceride accumulation. Similarly, ketogenic diets shift energy metabolism toward fatty acid oxidation and ketogenesis, reducing insulin levels and further downregulating DNL activity in the liver. The effects of time-restricted eating (TRE) on DNL primarily rely on the slight increase in ketone bodies and the alignment of circadian rhythms with metabolic processes [138], optimizing AMPK activity and reducing lipogenesis during fasting periods. This intervention also promotes lipid oxidation and improves overall metabolic flexibility. While evidence suggests that TRE impacts hepatic DNL more significantly than adipose tissue DNL, its effects on both tissues contribute to improved lipid profiles and reduced triglyceride accumulation. Collectively, these findings underscore the critical role of targeted lifestyle modifications in regulating DNL and improving metabolic health. By addressing the distinct contributions of liver and adipose tissue DNL, these interventions offer a comprehensive approach for managing lipid metabolism (Table 1). Further research is warranted to elucidate the long-term implications of these strategies and optimize their application across diverse populations.

One of the major limitations of research on de novo lipogenesis (DNL) in adipose tissue is the challenge of accurately determining the true origin of newly synthesized lipids within adipocytes. Specifically, it remains difficult to distinguish between lipids produced through intra-adipose tissue DNL and those originating from hepatic DNL, which are subsequently transported and stored in adipocytes. This methodological challenge significantly impacts the interpretation of findings related to lipid metabolism and storage.

In the context of this narrative review, these limitations impose significant constraints on available research. Specifically, it is difficult to discuss in detail the effects of dietary and lifestyle interventions—such as the ketogenic diet, time-restricted eating, and exercise—on adipose tissue DNL. The scarcity of dedicated studies addressing these interventions, combined with the challenge of differentiating the direct effects on adipocyte DNL from the indirect effects of hepatic lipid flux, makes it difficult to draw definitive conclusions. As a result, the ability to provide a comprehensive analysis of how these interventions modulate adipose tissue DNL is inherently limited by the current state of research. Resolving these methodological issues and developing more precise techniques to trace lipid origins would be essential for advancing our understanding of adipose tissue metabolism. Such advancements could not only refine future research but also improve clinical strategies for managing metabolic disorders linked to dysregulated lipogenesis and lipid accumulation.

## Figures and Tables

**Table 1:** Summary of the effects of physical exercise, ketogenic diets, and time-restricted eating (TRE) on de novo lipogenesis (DNL) and lipid metabolism. The table outlines the key metabolic pathways influenced by these interventions, including the regulation of lipogenic enzymes, lipid oxidation, mitochondrial function, and insulin sensitivity, with a focus on hepatic and adipose tissue DNL.

Topic	Key Points
De Novo Lipogenesis (DNL)	DNL is the process of converting excess carbohydrates into fatty acids, primarily occurring in the liver and, to a lesser extent, in adipose tissue. It is regulated by insulin and key transcription factors, including sterol regulatory element-binding protein 1c (SREBP-1c) and carbohydrate-responsive element-binding protein (ChREBP). These factors stimulate the expression of enzymes like acetyl-CoA carboxylase (ACC) and fatty acid synthase (FASN), which are essential for fatty acid synthesis.
Impact on Metabolic Health	Excessive hepatic DNL leads to triglyceride accumulation within hepatocytes, contributing to metabolic-associated steatotic liver disease (MASLD). In contrast, adipose DNL is limited and more dependent on local substrate availability rather than systemic carbohydrate intake. The primary contributors to adipose fat storage are dietary fat uptake and re-esterification of free fatty acids rather than newly synthesized fatty acids from DNL.
Physical Exercise and DNL	Physical activity, particularly endurance and resistance training, reduces substrate availability for DNL by increasing glucose uptake in skeletal muscle and lowering circulating insulin levels. Exercise also activates AMP-activated protein kinase (AMPK), which inhibits SREBP-1c, downregulating ACC and FASN activity. Additionally, PPARγ coactivator-1α (PGC-1α), a key regulator of mitochondrial biogenesis, enhances fatty acid oxidation, further reducing hepatic triglyceride accumulation.
Ketogenic Diet and DNL	The ketogenic diet shifts metabolism toward fatty acid oxidation and ketogenesis, leading to reduced insulin signaling and subsequent downregulation of SREBP-1c and ChREBP. This suppression decreases the expression of lipogenic enzymes such as ACC and FASN, inhibiting DNL. Additionally, the ketogenic diet increases circulating β-hydroxybutyrate (BHB), which has been shown to inhibit histone deacetylases (HDACs) and modulate gene expression, favoring lipid oxidation over synthesis.
Time-Restricted Eating (TRE) and DNL	TRE aligns metabolic processes with circadian rhythms, optimizing AMPK activity during fasting periods. This activation leads to suppression of lipogenesis and an increase in fatty acid oxidation via PPARα activation. Additionally, TRE enhances mitochondrial function and increases levels of fibroblast growth factor 21 (FGF21), which plays a key role in lipid metabolism by promoting fatty acid β-oxidation and reducing endoplasmic reticulum (ER) stress, thereby further inhibiting hepatic DNL.
Molecular Mechanisms	The combined effects of exercise, ketogenic diets, and TRE regulate DNL through multiple interconnected pathways: (1) AMPK activation suppresses SREBP-1c and ChREBP, reducing lipogenic enzyme activity; (2) PGC-1α enhances mitochondrial biogenesis and fatty acid oxidation; (3) FGF21 modulates lipid metabolism and insulin sensitivity; (4) BHB inhibits HDACs, reducing hepatic lipogenesis and inflammation. These mechanisms collectively decrease hepatic triglyceride accumulation, improve insulin sensitivity, and promote overall metabolic flexibility.

## Data Availability

Not applicable.

## Abbreviations

The following abbreviations are used in this manuscript:

ACC	acetyl-CoA carboxylase
AMPK	AMP-activated protein kinase
ChREBP	carbohydrate-responsive element-binding protein
DNL	de novo lipogenesis
FAS	fatty acid synthase
FGF21	fibroblast growth factor
IHTG	intrahepatic triglyceride
MASLD	metabolic dysfunction-associated steatotic liver disease
NEFA	non-esterified fatty acids
PPARα	peroxisome proliferator-activated receptor alpha
SREBP-1c	sterol regulatory element-binding protein-1c
TRE	time-restricted eating
